# Usefulness of phase angle on bioelectrical impedance analysis as a surveillance tool for postoperative infection in critically ill patients

**DOI:** 10.3389/fmed.2023.1111727

**Published:** 2023-02-22

**Authors:** Gyeo Ra Lee, Eun Young Kim

**Affiliations:** Division of Trauma and Surgical Critical Care, Department of Surgery, Seoul St. Mary’s Hospital, College of Medicine, The Catholic University of Korea, Seoul, Republic of Korea

**Keywords:** infection, inflammatory marker, intensive care unit, phase angle, surgery

## Abstract

**Purpose:**

Bioelectrical impedance analysis (BIA) has advantages of obtaining results quickly, safely, reproducibly, and non-invasively. Phase angle (PhA) is one of the parameter of BIA, its values represent the permeability or integrity of cell membrane. With the exception of C-reactive protein (CRP), few studies have estimated an association between PhA and these conventional biomarkers. Herein, we aimed to investigate the association between the PhA value and the conventional inflammatory markers in postoperative patients in intensive care unit (ICU). Also, the correlation between the change in PhA and the occurrence of infectious complication were determined.

**Methods:**

From July 2020 to February 2022, retrospective observation study conducted in 221 patients who admitted to ICU after abdominal surgery. BIA measurements and blood sampling were routinely performed the next morning. The relationship between PhA and the inflammatory markers were assessed after adjusting for age and body mass index. Univariate and multivariate logistic regression analysis was performed to examine the predisposing factors for postoperative infections.

**Results:**

Among 221 patients admitted to ICU after abdominal surgery, infectious complications occurred in 62 cases. CRP, procalcitonin, or presepsin levels were negatively correlated with PhA in both gender. (−0.295, −0.198 or −0.212 of partial correlation coefficients, respectively in males, and 0.313, −0.245 or −0.36 of partial correlation coefficients, respectively in females) But, white blood cell did not show significant association with PhA in both genders. For males, increased level of CRP on postoperative day 1 (POD1) was revealed as the significant predicting factor for postoperative infectious complication [odds ratio (OR): 1.184, 95% confidence interval (CI): 1.090–1.285, *p* < 0.001]. For females, increased Acute Physiology and Chronic Health Evaluation II score at admission (OR: 1.457, 95% CI: 1.068–1.987, *p* = 0.018), increased level of presepsin on (OR: 1.003, 95% CI: 1.001–1.006, *p* = 0.016) and decreased value of PhA on POD1 (OR: 0.980, 95% CI: 0.967–0.993, *p* = 0.003) were revealed as the significant predicting factors.

**Conclusion:**

Phase angle obtained through BIA can be used as a predictor of infection as it shows a significant association with inflammatory markers. Phase angle measurements through BIA could improve patient prognosis after abdominal surgery through the careful observation of infections and early, appropriate treatment.

## Introduction

Postoperative infections frequently occur in patients who undergo abdominal surgery, (1, 2) and its rate is relatively high, ranging from about 20% to 40%. Various conventional markers such as C-reactive protein (CRP) and white blood cell (WBC) counts are commonly used to detect infections in clinical practice, and recently, some novel markers such as procalcitonin (PCT) and presepsin were also proposed. However, those markers need blood sampling to obtain the results, and the results cannot be confirmed in real-time. Additionally, there is a decisive limitation in that it is impossible to perform the laboratory tests of these markers in all hospitals because special equipment and professional personnel are essential to obtaining the results.

Bioelectrical impedance analysis (BIA) measures the resistance and reactance of body components by recording the voltage drop according to a given electric current (3, 4). The phase angle (PhA) is a parameter obtained from BIA through the relationship between the reactance and resistance of body tissues and represents the permeability or integrity of the cell membranes as a biological marker of cellular health (5, 6). A previous study reported that lower PhA values in critically ill hospitalized patients were associated with increased mortality rates and complications (7, 8). Pena et al. (9), Barros et al. (10), and Roccamatisi et al. (11) reported that low preoperative PhA values measured by BIA were associated with increased rates of infections after elective surgery. Several studies have reported a relationship between the PhA and various inflammatory markers (12–17). Moreover, the PhA from BIA is more advantageous than conventional markers as a diagnostic tool because the results are obtained safely, non-invasively, and more quickly. However, few studies have estimated an association between the PhA and conventional markers in postoperative patients who are vulnerable to infectious complication.

Herein, we aimed to investigate the association between the PhA value obtained through BIA surveillance and the conventional inflammatory markers in the acute phase of postoperative patients in the intensive care unit (ICU). Also, the relationship between changes in the PhA value during the postoperative period and the occurrence of infections was determined.

## Materials and methods

### Study design and patient enrollment

From July 2020 to February 2022, we performed a prospective observational study in a 22-bed ICU of a single tertiary hospital. All patients aged over 18 years who were admitted to the ICU after abdominal surgery performed under general anesthesia were eligible for inclusion, regardless of the surgical technique, such as laparotomy, laparoscopic or robotic surgery. If the patient underwent endovascular surgery or percutaneous transluminal angioplasty, they were excluded from enrollment. However, cases of open thrombectomy or emergent exploration due to abdominal aortic aneurysmal rupture were enrolled. Patients who met any one of these criteria were excluded from study enrollment: (1) those who had any contraindication or significant confounders of BIA, such as any prosthetic medical devices including an implanted cardiac defibrillator, pacemaker, or metallic intravascular device, or any bone fixation implants or limb amputation; (2) pregnant women; (3) those who underwent extracorporeal membrane oxygenation treatment before surgery; (4) those who were readmitted within 48 h after discharge from the ICU or died within 72 h after surgery; (5) those who were admitted to the ICU only for medical causes without surgery, and (6) those lacking or missing essential BIA data. Patients such as those receiving hemodialysis for end-stage renal disease or severe acute kidney injury that could be significant confounders of laboratory inflammation tests such as presepsin (18, 19), were also excluded from the study analysis. Written informed consent was obtained from each patient and the recruiting data, including demographics, disease profile, and laboratory results, were reviewed retrospectively. This study was approved and carefully monitored by our Institutional Review Board (No. IRB; KC22RISI0346), and was performed in accordance with the 1964 Declaration of Helsinki and its later amendments.

### BIA measurement

**Figure 1:**
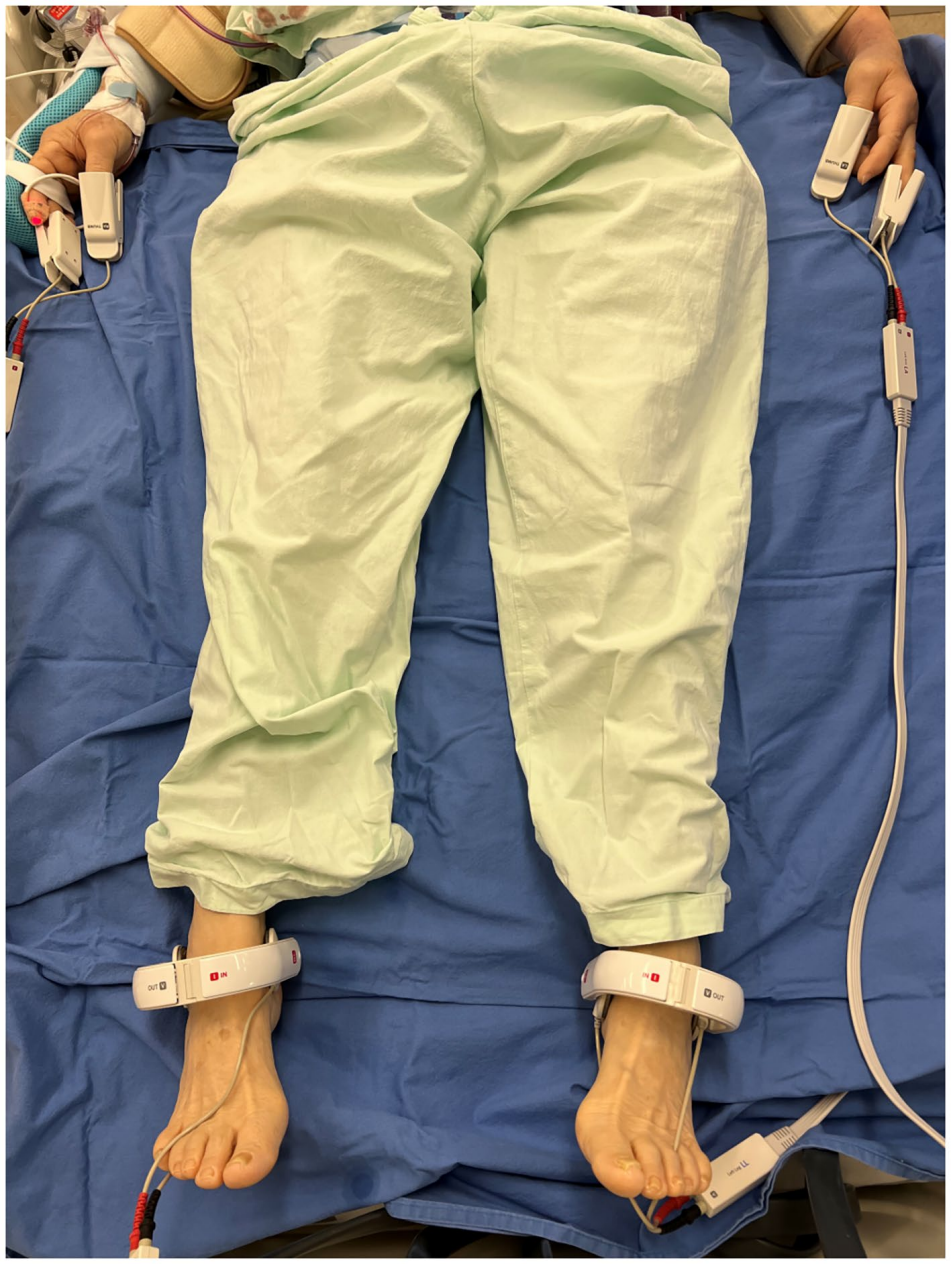
Electrodes placement for bioelectrical impedance analysis.

BIA measurements were routinely performed the morning after the patients were admitted postoperatively to the ICU. The body composition status was assessed using a commercial portable BIA device with 50-kHz alternating current (InBody S10®, InBody Corp., Seoul, Korea), (20) and was designed using touch- or adhesive-type electrodes attached to four limbs, as described in a study of Lee et al. (20) (Figure 1). This device has an intrinsic impedance of 1.6 Ω when measured at 50 kHz. However, since the electrode-skin contact impedance appears very different depending on the skin condition, it is difficult to calculate an absolute value or absolute range. All participants had refrained from eating or drinking for 6 hours before BIA measurement. And, participants are placed in a supine position, with the extremities in a relaxed position. Two pairs of electrodes were placed, hand electrode are inserted to thumb and middle finger, and foot electrodes are placed between the malleolus and the heel. Regarding the environmental condition that could affect the BIA value, the ICU of our institution is specially controlled to maintain a relatively constant environment at a temperature of 24°C and humidity between 35% and 40% (21). To minimize measurement errors, BIA measurements were performed by the same well-trained physician and supervised by another senior physician. The time required for BIA measurement was about 2 min for each patient, and the body composition status data were immediately analyzed and printed in real-time. For each patient, the following BIA parameters were obtained: intracellular water, extracellular water (ECW), total body water, ECW ratio, which was defined as the ratio of ECW to total body water, whole-body and segmental PhA, impedance, and reactance. PhA was defined as the physiological index of cell membrane integrity and vitality to reflect the quantity and quality of the soft tissues. In general, a higher PhA value indicates greater cellularity, cellular function, and cell membrane integrity (10). It was calculated using the following formula:


$$Ø=\arctan\left(57.296\;\;\times \;\;X_{c}/R\right)$$

(where *Ø* is the phase angle, *X*_c_ is reactance, and *R* is resistance). The coefficient of variation (CV) of repeated *R* and *X*_c_ measurements at 50 kHz was assessed in 10 patients (7 males and 3 females) by the same physician. The CVs were 0.37% for *R* and 1.49% for Xc.

### Other data variables and clinical outcome assessment

Laboratory tests on blood samples obtained from all participants were routinely conducted at the same time as the BIA measurements, and inflammatory markers such as CRP, PCT, and presepsin were also measured. The following data were obtained from the electronic medical records: demographics, surgical profiles, including the site of surgery, disease characteristics, and severity index using Acute Physiology and Chronic Health Evaluation II (APACHE II) and sequential organ failure assessment (SOFA) scores at ICU admission, and the presence of shock. Any development of postoperative morbidities during hospitalization was monitored and recorded. Postoperative morbidities were classified from grade 0 to 5 according to the *Clavien-Dindo* classification (22). According to the definition by the “Bulletin of the American College of Surgeons,” (23) infection complications included operative wound dehiscence with openings greater than 3 cm, surgical site infections defined by the Centers for Disease Control guidelines for the prevention of SSI, pneumonia, bacteremia, urinary tract infection, sepsis or septic shock, and a sustained fever over 38°C with infection. A simple hematoma or seroma, such as an SSI that did not require any additional treatment, was not considered an infection-related morbidity. Postoperative mortality was defined as mortality within 30 days of surgery or within the same hospitalization as the surgery.

### Statistical analysis

All statistical analyses were conducted using SPSS statistical package software (version 24.0 for Windows; SPSS, Inc., Chicago, IL, USA). Continuous data are presented as the mean ± standard deviation, and the overall differences were tested by the Student’s *t*-test or analysis of variance. Whether the variables were normally distributed was tested using the *Kolmogorov–Smirnov* test, and in the case of variables that were not normally distributed, a nonparametric test was performed using the *Mann–Whitney* test. The sample size was obtained through the Bland & Altman method. The probability of rejecting the null hypothesis as known as a type I error was set to 0.05, and the probability of accepting the null hypothesis when in fact it is false as known as type II error was set to 0.20. For males, a minimum required size of calculated sample was smaller than our recruited population of 132, and for females, it was smaller than our population of 89. The categorical variables were calculated using Fisher’s exact test or Chi-squared (*χ*^2^) test. The relationship between the PhA on BIA and inflammatory markers such as CRP, PCT, or presepsin measured by blood tests, was assessed using Pearson’s correlation coefficients (r) after adjusting for age and body mass index (BMI). Linear regression analysis was used to assess the relationship between the PhA on BIA and inflammatory markers, and the Bland–Altman plot was used to assess the agreement between those parameters. The differences were regarded as statistically significant for *p*-values <0.05. Differences in the incidence of postoperative infections were analyzed according to the PhA value on BIA measured postoperative day 1 (POD1). Univariate logistic regression analysis was performed to examine the predisposing factors of postoperative infections, and based on these, the significantly correlated variables were analyzed by multivariate logistic regression.

## Results

**Figure 2:**
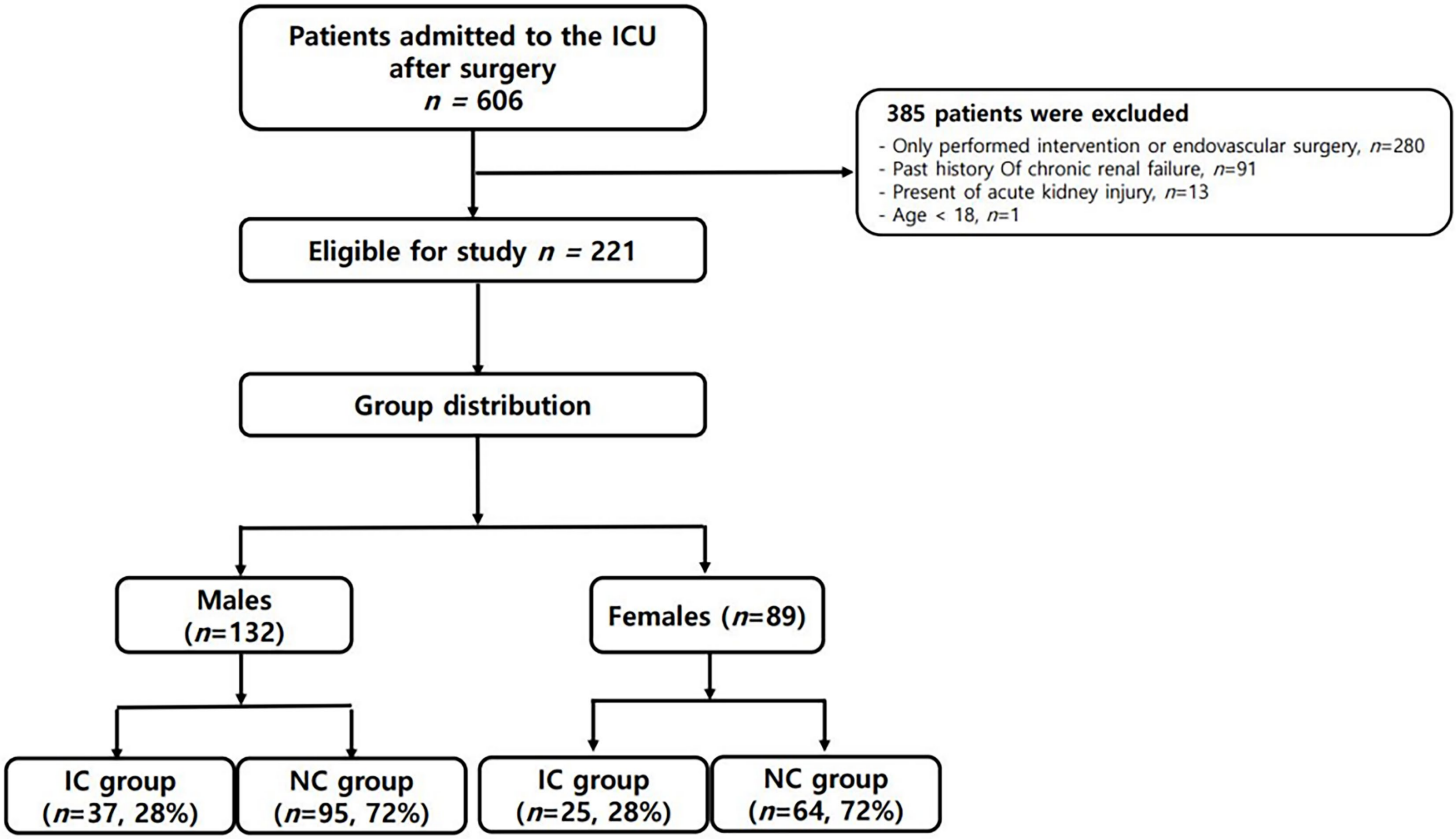
Schematic diagram of study enrollment. *IC group: group with infections; ICU: intensive care unit; NC group: group without infections.

During the study period, a total of 606 patients were admitted to the ICU after abdominal surgery. As shown in Figure 2, a total of 221 patients were finally analyzed according to our inclusion criteria. Given that the normal reference range of BIA data differs according to gender, we divided the patients into males (132 patients, 59.7%) and females (89 patients, 40.3%), and analyzed the results, respectively. The patients were also subdivided into groups with infections (IC group) and without infections (NC group). As shown in Table 1, there was no significant difference in age, BMI, or underlying disease between the IC group and the NC group in either gender. For females, disease severity represented by SOFA score or APACHE II score was significantly higher in the IC group than in the NC group (*p* = 0.001 and *p* < 0.001, respectively). Regarding the inflammatory markers measured on the first POD1, the WBC count did not differ between the IC group and the NC group. However, CRP and PCT levels were significantly higher in the IC group than in the NC group. Presepsin levels were also significantly higher in the IC group of females. In the comparison of BIA measurements, in males, there were no significant differences between the two groups, but the PhA was slightly lower in the IC group than in the NC group (4.22 ± 1.92 vs. 4.68 ± 1.07, *p* = 0.085). The *value of p* was greater than 0.05 but within the upper limit of 0.1. In females, the PhA was significantly lower in the IC group than in the NC group (3.18 ± 1.02 vs. 4.25 ± 1.02, *p* < 0.001). Also, ECW and the ECW ratio were significantly higher in the IC group. Regarding clinical outcomes, the length of hospital stay was significantly longer in the IC group of males. In females, the length of mechanical ventilation, length of ICU stay and length of hospital stay was significantly longer in the IC group.

**Table 1:** Comparative analysis of demographic characteristics and clinical outcomes between two groups according to occurrence the infection after surgery by dividing into males and females.

Infections	Males (*n* = 132, 59.7%)			Females (*n* = 89, 40.3%)		
	+ (*n* = 37, 28%)	− (*n* = 95, 72%)	*p*-value	+ (*n* = 25, 28%)	− (*n* = 64, 72%)	*p*-value
Baseline characteristics						
Age, years (median, IQR)	66.7 [39–96]	67.7 [22–90]	0.680	69.7 [41–94]	63.2 [25–92]	0.071
BMI (kg/m^2^)	23.6 ± 4.2	23.1 ± 3.2	0.551	22.1 ± 3.9	23.1 ± 3.3	0.249
Subjective global assessment score	1.41 ± 0.59	1.34 ± 0.58	0.553^*^	1.26 ± 0.45	1.25 ± 0.51	0.914^*^
Underlying disease, *n* (%)						
Solid tumor/malignancy	22 (59.5%)	69 (72.6%)	0.149	14 (56%)	42 (65.6%)	0.467
Diabetes mellitus	9 (24.3%)	26 (27.4%)	0.828	4 (16%)	14 (21.9%)	0.770
COPD	1 (2.7%)	6 (6.3%)	0.405	-	-	-
Chronic liver disease	1 (2.7%)	3 (3.2%)	0.891	1 (4%)	1 (1.6%)	0.486
Disease severity						
SOFA score (mean, ±SD)	2.4 ± 2.6	2.7 ± 2.4	0.610^*^	3.6 ± 3.4	1.8 ± 1.5	0.001^*^
APACHE II (mean, ±SD)	11.1 ± 4.7	9.5 ± 4	0.077^*^	12.9 ± 5.3	8.4 ± 3.8	<0.001^*^
Site of surgery, *n* (%)						
Gastrointestinal surgery	24 (64.9%)	29 (30.5%)	0.001	20 (80%)	19 (29.7%)	<0.001
Hepatobiliary surgery	12 (32.4%)	57 (60%)	0.006	4 (16%)	37 (57.8%)	<0.001
Vascular surgery	1 (2.7%)	8 (8.4%)	0.242	-	-	*-*
Trauma or miscellaneous	-	1 (1.1%)	0.531	1 (4%)	8 (12.5%)	0.232
Clinical outcomes						
Length of mechanical ventilation, day	0.4 ± 1.4	0.1 ± 0.6	0.110^*^	0.8 ± 1.7	0	<0.001^*^
Length of ICU stay, day	2.4 ± 4.7	1.5 ± 1.2	0.07^*^	2.5 ± 2.4	1.4 ± 1.1	0.002^*^
Length of hospital stay, day	23.3 ± 24.7	16 ± 9.5	0.015^*^	29 ± 24.3	14.4 ± 11.9	<0.001*
ICU mortality, *n* (%)	-	1 (1.1%)	0.531	-	-	-
In-hospital mortality, *n* (%)	-	2 (2.1%)	0.374	1 (4%)	-	0.108
Postoperative complication						
Infections, *n* (%)	37 (100%)	-	*-*	25 (100%)	*-*	*-*
Pneumonia	5 (13.5%)	-	-	4 (16%)	*-*	*-*
Intra-abdominal infection	33 (89.2%)	-	-	21 (84%)	*-*	*-*
Wound infection	-	-	*-*	-	-	*-*
Non-infectious complication, *n* (%)	4 (10.8%)	8 (8.4%)	0.668	2 (8%)	4 (6.3%)	0.767
Tachyarrhythmia	1 (2.7%)	1 (1.1%)	0.486	-	2 (3.1%)	0.371
Pleural effusion	3 (8.1%)	5 (5.3%)	0.686	2 (8%)	1 (1.6%)	0.130
Postoperative bleeding	-	4 (4.2%)	0.205	-	1 (1.6%)	0.530

APACHE II: acute physiology and chronic health evaluation II, BMI: body mass index, COPD: chronic obstructive pulmonary disease, SD: standard deviation, SOFA: sequential organ failure assessment.

^*^For non-normally distributed variables, non-parametric tests were performed using the Mann–Whitney test.

### Relationship between the PhA measured at 50 kHz on BIA and various parameters

**Figure 3:**
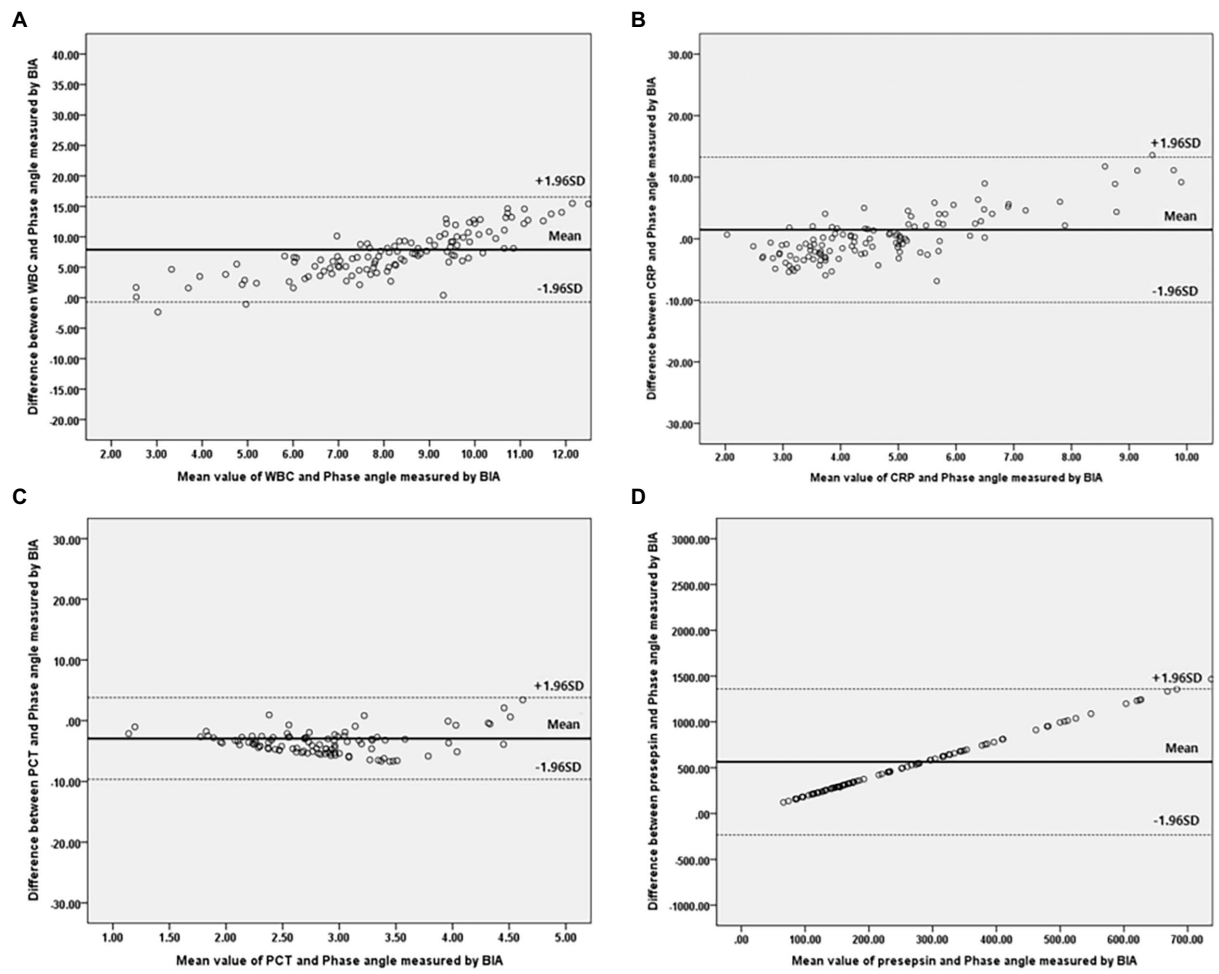
Bland–Altman plot for analyzing the agreement between the inflammatory marker and Phase angle on BIA in males. **(A)** White blood cell, **(B)** C-reactive protein, **(C)** Procalcitonin, **(D)** Presepsi. *BIA: bioelectrical impedance analysis.

**Figure 4:**
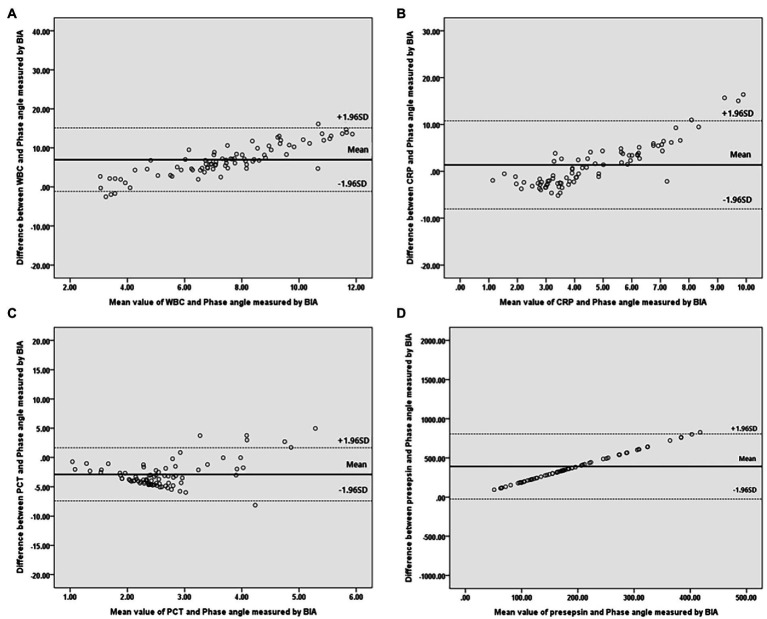
Bland–Altman plot for analyzing the agreement between the inflammatory marker and Phase angle on BIA in females. **(A)** White blood cell, **(B)** C-reactive protein, **(C)** Procalcitonin, **(D)** Presepsi. *BIA: bioelectrical impedance analysis.

Bivariate correlation analysis was performed on the association between PhA measured at 50 kHz on BIA and various variables by gender, and the correlations between the variables were similar regardless of gender. Firstly, anthropometric parameters, such as BMI, were positively correlated with the PhA in both genders, whereas age was negatively correlated with the PhA. Additionally, inflammatory markers, such as CRP, PCT, and presepsin levels, were also negatively correlated with the PhA (−0.262, −0.22, and −0.22 correlation coefficients, respectively, in males, and −0.361, −0.277, and −0.347 correlation coefficients, respectively, in females). WBC counts were not associated with PhA values. After adjusting for age and BMI, a partial correlation analysis was performed, as shown in Table 2. CRP, PCT, and presepsin levels were negatively correlated with the PhA in both genders (−0.295, −0.198, and −0.212 partial correlation coefficients, respectively, in males, and 0.313, −0.245, and −0.36 partial correlation coefficients, respectively, in females). WBC counts were not significantly associated with the PhA in either gender. Also, compared inflammatory markers and phase angle on BIA through the Bland and Altman plot (Figures 3, 4). All of them were distributed between the 95% limit of agreement. For all variables except for presepsin, it was uniformly distributed around the bias, the plot shows a significant correlation between inflammatory marker and phase angle on BIA. Only presepsin showed an upward sloping pattern, but since there are values between the 95% limit of agreement limit, it is impossible to assured that there is no correlation.

**Table 2:** Partial correlation between PhA measured at 50 kHz on BIA and various parameters after adjustment for age and BMI.

Parameters	Males (*n* = 132, 59.7%)		Females (*n* = 89, 40.3%)	
	Partial correlation coefficients	*p*-value	Partial Correlation Coefficients	*p*-value
Inflammatory marker^a^				
WBC	−0.108	0.222	−0.024	0.828
C-reactive protein	−0.295	0.001	−0.313	0.003
Procalcitonin	−0.198	0.024	−0.245	0.022
Presepsin	−0.212	0.015	−0.360	0.001

BIA: bioelectrical impedance analysis, BMI: body mass index, kHz: kilohertz, PhA: phase angle, WBC: white blood cell.

a Laboratory markers and BIA data performed on the 1^st^ day after surgery.

### Determination of predictive factors of postoperative infections

Table 3 demonstrates the results of logistic regression analysis for infections that occurred after surgery in males. After univariate analysis, the increased CRP levels on POD1 and increased PCT levels on POD1 were significant predictive factors for infections in males admitted to the ICU after surgery. After multivariate analysis, only increased levels of CRP on POD1 were revealed as significant predictive factors for postoperative infections in males [odds ratio(OR) = 1.184, 95% confidence interval (CI): 1.090–1.285, *p* < 0.001]. In females, as shown in Table 4, increased SOFA scores and APACHE II scores at admission, and increased CRP, PCT, and presepsin levels on POD1 were significant factors in univariate analysis. Decreased PhA values on POD1 (OR = 0.354, 95% CI: 0.206–0.608, *p* < 0.001) were significant factors in univariate analysis. After multivariate analysis, increased APACHE II scores at admission (OR = 1.457, 95% CI: 1.068–1.987, *p* = 0.018), increased presepsin levels on POD1 (OR = 1.003, 95% CI: 1.001–1.006, *p* = 0.016), and decreased PhA values on POD1 (OR = 0.980, 95% CI: 0.967–0.993, *p* = 0.003) were revealed as significant predictive factors for post-surgical infections in females.

**Table 3:** Predictors of postoperative infections in patients who underwent abdominal surgery by univariate and multivariate logistic regression analysis in *males*.

Parameters	Univariate analysis		Multivariate analysis	
	OR (95% CI)	*p*-value	OR (95% CI)	*p*-value
Age	0.993 (0.964–1.024)	0.670		
Underlying disease				
DM	0.853 (0.355–2.048)	0.722		
Solid tumor/Malignancy	0.553 (0.249–1.225)	0.144		
Chronic liver disease	0.852 (0.086–8.460)	0.891		
Disease severity				
SOFA score	1.006 (0.850–1.192)	0.942		
APACHE II	1.091 (0.997–1.193)	0.158		
Inflammatory marker^a^				
WBC	0.957 (0.896–1.023)	0.196		
C-reactive protein	1.194 (1.103–1.293)	<0.001	1.184 (1.090–1.285)	<0.001
Procalcitonin	1.030 (1.005–1.056)	0.02	1.010 (0.984–1.036)	0.461
Presepsin	1.000 (0.999–1.001)	0.875		
Body composition parameter^a^				
PhA	0.763 (0.559–1.040)	0.087	0.971 (0.705–1.337)	0.857

Postoperative infections: According to the definition by the “Bulletin of the American College of Surgeons,” the infectious complications included operative wound dehiscence with openings greater than 3 cm, surgical site infections (SSI) defined by the Centers for Disease Control (CDC) guidelines for the prevention of SSI, pneumonia, bacteremia, urinary tract infection, sepsis or septic shock and a sustained fever over 38°C with infection. A simple hematoma or seroma, which did not require any additional treatment, was not considered as an infectious morbidity such as SSI.

APACHE II: acute physiology and chronic health evaluation II, CI: confidence interval, DM: diabetes mellitus, OR: odds ratio, PhA: phase angle, SOFA: sequential organ failure assessment, WBC: white blood cell.

a Laboratory markers and BIA data performed on the 1^st^ day after surgery.

**Table 4:** Predictors of postoperative infections in patients who underwent abdominal surgery by univariate and multivariate logistic regression analysis in females.

Parameters	Univariate analysis		Multivariate analysis	
	OR (95% CI)	*p*-value	OR (95% CI)	*p*-value
Age	1.032 (0.997–1.068)	0.172		
Underlying disease				
DM	0.680 (0.200–2.310)	0.537		
Solid tumor/Malignancy	0.667 (0.260–1.712)	0.399		
Chronic liver disease	2.625 (0.158–43.662)	0.501		
Disease severity				
SOFA score	1.481 (1.133–1.936)	0.004	0.638 (0.333–1.224)	0.177
APACHE II	1.252 (1.105–1.419)	<0.001	1.457 (1.068–1.987)	0.018
Inflammatory marker^a^				
WBC	0.930 (0.842–1.027)	0.153		
C-reactive protein	1.201 (1.092–1.320)	<0.001	1.155 (0.989–1.349)	0.068
Procalcitonin	1.529 (1.135–2.060)	0.005	1.142 (0.904–1.444)	0.266
Presepsin	1.002 (1.001–1.003)	0.002	1.003 (1.001–1.006)	0.016
Body composition parameter^a^				
PhA	0.354 (0.206–0.608)	<0.001	0.980 (0.967–0.993)	0.003

Postoperative infections: According to the definition by the “Bulletin of the American College of Surgeons,” the infectious complications included operative wound dehiscence with openings greater than 3 cm, surgical site infections (SSI) defined by the Centers for Disease Control (CDC) guidelines for the prevention of SSI, pneumonia, bacteremia, urinary tract infection, sepsis or septic shock and a sustained fever over 38°C with infection. A simple hematoma or seroma, which did not require any additional treatment, was not considered as an infectious morbidity such as SSI.

APACHE II: acute physiology and chronic health evaluation II, CI: confidence interval, DM: diabetes mellitus, OR: odds ratio, PhA: phase angle, SOFA: sequential organ failure assessment, WBC: white blood cell.

a Laboratory markers and BIA data performed on the 1^st^ day after surgery.

## Discussion

Our results showed that the PhA on BIA was strongly negatively correlated with CRP, PCT, and presepsin in both males and females who underwent abdominal surgery. In multivariate analysis, decreased PhA values on POD1, increased APACHE II scores at admission, and increased presepsin levels on POD1 were revealed as significant predictors of post-surgical infections in females. In males, only increased CRP levels on POD1 were a significant predictor of infections.

BIA is a tool that can indirectly identify cellular damage by assessing the quality of whole-body cell membranes. Among the parameters of BIA, the PhA has been reported to be closely related to the degree of inflammation *in vivo* (6, 24). Oxidative stress resulting from an imbalance between oxidants and antioxidants, with increased reactive oxygen species, occurs in severe inflammation. It can lead to cellular injury by damaging cellular components such as proteins or lipids. These alterations can cause the cellular membrane to rupture, and the breakdown of the membrane’s phospholipid structure causes the transformation of cell shape and fluid imbalance by promoting the migration of intracellular water molecules to the extracellular environment. As a result, the ECW ratio is increased due to a fluid shift from intra cellular water to ECW, and this causes a decrease in cell mass that eventually leads to a decrease in the PhA value. Thus, a high PhA value might indicate a high proportion of healthy cell membranes, and conversely, low PhA could be associated with cell death or decreased cell integrity (25, 26). In fact, the low PhA observed after surgery that associated with cell loss and cell integrity was also described in the study of Petro et al. (27). Therefore, low PhA values are expected to sensitively detect oxidative stress, which is closely related to inflammation. It is noteworthy that oxidative stress may be more pronounced in infectious environments with severe inflammation, where typically, the host’s metabolic response is increased, resulting in the increased production of oxygen metabolites. In fact, our results also showed a significant correlation between PhA values and various inflammatory markers, such as CRP, PCT, and presepsin, commonly used to detect infections in clinical practice. As shown in Table 5, PhA values were significantly lower in the IC group with postoperative infections compared to the NC group without infections, regardless of gender. Additionally, in univariate analysis, PhA values were negatively associated with postoperative infections in both genders, and these values were a significant predictor of post-surgical infections in females. Therefore, we expect that the PhA value on BIA could be useful as a monitoring tool to detect infections accompanied by severe inflammation after abdominal surgery, where oxidative stress is increased due to severe inflammation and enhancement of the immune response and metabolic processes in the body.

**Table 5:** Comparative analysis of laboratory markers and BIA data between two groups according to occurrence the infection after surgery by dividing into males and females.

POD 1	Males (*n* = 132, 59.7%)			Females (*n* = 89, 40.3%)		
	+ (*n* = 37, 28%)	− (*n* = 95, 72%)	*p*-value	+ (*n* = 25, 28%)	− (*n* = 64, 72%)	*p*-value
Laboratory markers						
WBC * 10^3^/mL	12.38 ± 7.42	14.32 ± 7.67	0.186^a^	10.38 ± 6.33	12.29 ± 5.33	0.189^a^
C-reactive protein (mg/dl)	12.07 ± 9.57	4.68 ± 3.65	<0.001^a^	12.77 ± 10.16	4.91 ± 3.65	<0.001^a^
Procalcitonin (ng/mL)	12.32 ± 19.44	4.03 ± 14.01	0.007^a^	18.72 ± 33.46	0.87 ± 1.26	<0.001^a^
Presepsin (pg/mL)	731.6 ± 465.6	711.4 ± 731.9	0.851^a^	1,253.8 ± 1,754.5	433.4 ± 322.2	<0.001^a^
BIA data						
Phase angle _ WB (°)	4.36 ± 1.29	4.63 ± 1.4	0.288^a^	3.18 ± 1.02	4.25 ± 1.02	<0.001^a^
Phase angle _ RA (°)	4.52 ± 3.75	4.33 ± 1.32	0.775^a^	3.1 ± 0.88	3.65 ± 0.74	0.009
Phase angle _ LA (°)	3.83 ± 1.08	4.17 ± 1.21	0.13^a^	2.89 ± 0.68	3.65 ± 0.62	<0.001^a^
Phase angle _ TR (°)	3.88 ± 1.49	4.12 ± 1.68	0.428	3.44 ± 1.27	3.92 ± 1.41	0.128
Phase angle _ RL (°)	4.65 ± 1.32	5.18 ± 2.01	0.08^a^	3.24 ± 1.49	5.15 ± 2.21	<0.001^a^
Phase angle _ LL (°)	4.37 ± 1.46	5.09 ± 1.99	0.024^a^	3.11 ± 1.42	5.03 ± 2.19	<0.001^a^
ECW (L)	14.6 ± 2.6	15.3 ± 2.5	0.207^a^	12.5 ± 1.7	11.5 ± 1.6	0.023
ICW (L)	22.1 ± 4.7	23.5 ± 4.2	0.124^a^	17.6 ± 2.1	17.7 ± 2.5	0.870
ECW ratio	0.39 ± 0.02	0.39 ± 0.01	0.194	0.41 ± 0.02	0.39 ± 0.02	<0.001^a^
At 36 h postoperatively	+ (*n* = 18, 41.9%)	− (*n* = 25, 58.1%)	*p*-value	+ (*n* = 8, 40%)	− (*n* = 12, 60%)	*p*-value
Laboratory markers						
WBC * 10^3^/mL	11.67 ± 4.99	13.56 ± 5.12	0.063^a^	12.62 ± 6.77	12.31 ± 6.42	0.914^a^
C-reactive protein (mg/dl)	16 ± 7.95	14.33 ± 6.16	0.202	13.93 ± 7.64	10.71 ± 5.62	0.059^a^
Procalcitonin (ng/mL)	9.74 ± 20.5	15.65 ± 110.35	0.181^a^	9.28 ± 22.48	0.61 ± 0.85	0.173^a^
Presepsin (pg/mL)	805.11 ± 897.57	719.61 ± 568.7	0.745^a^	875.27 ± 1161.84	484.45 ± 309.3	0.217^a^
BIA data						
Phase angle _ WB (°)	3.79 ± 1.17	4.39 ± 1.31	0.123	3.74 ± 0.73	4 ± 0.72	0.437
Phase angle _ RA (°)	3.84 ± 1.16	4.3 ± 1.22	0.215	3.14 ± 0.83	3.6 ± 0.64	0.173^a^
Phase angle _ LA (°)	3.69 ± 1.18	3.98 ± 1.11	0.433	3.29 ± 0.46	3.48 ± 0.53	0.405
Phase angle _ TR (°)	3.61 ± 1.97	3.66 ± 1.72	0.928	3.46 ± 1.29	3.44 ± 1.49	0.998
Phase angle _ RL (°)	3.74 ± 1.36	4.8 ± 1.94	0.42	3.93 ± 0.85	4.83 ± 1.18	0.080
Phase angle _ LL (°)	3.82 ± 1.32	4.62 ± 1.69	0.087	3.9 ± 1.04	4.68 ± 1.11	0.134
ECW (L)	15.9 ± 2	15.1 ± 2.5	0.209	12.4 ± 1.5	11.7 ± 2	0.377
ICW (L)	23.4 ± 3.9	22.6 ± 3.7	0.503	17.3 ± 1.7	17.9 ± 3	0.515
ECW ratio	0.41 ± 0.01	0.4 ± 0.02	0.157	0.41 ± 0.01	0.39 ± 0.01	0.151
At 72 h postoperatively	+ (*n* = 4, 44.4%)	− (*n* = 5, 55.6%)	*p*-value	+ (*n* = 2, 50%)	− (*n* = 2, 50%)	*p*-value
Laboratory markers						
WBC * 10^3^/mL	10.28 ± 5.07	12.3 ± 5.98	0.034^a^	11.78 ± 6.77	10.22 ± 5.11	0.407^a^
C-reactive protein (mg/dL)	12.87 ± 6.59	13.61 ± 6.54	0.571	13.98 ± 9.98	9.36 ± 4.78	0.115^a^
Procalcitonin (ng/mL)	6.11 ± 12.88	15.24 ± 114.57	0.496^a^	6.41 ± 15.58	0.38 ± 0.5	0.229
Presepsin (pg/mL)	817.6 ± 722.9	866 ± 773.9	0.741^a^	1,059.67 ± 916.37	592.18 ± 413.51	0.025^a^
BIA data						
Phase angle _ WB (°)	2.32 ± 0.52	3.4 ± 0.58	0.053	2.68 ± 0.72	2.8 ± 0.7	0.859
Phase angle _ RA (°)	2.38 ± 0.44	3.06 ± 1.25	0.303	2.72 ± 0.82	2.25 ± 1.34	0.709
Phase angle _ LA (°)	2.55 ± 0.26	2.96 ± 0.86	0.359	2.44 ± 0.96	2.8 ± 0.57	0.577
Phase angle _ TR (°)	2.65 ± 0.92	3.24 ± 1.28	0.448	2.92 ± 0.82	3.2 ± 1.28	0.811
Phase angle _ RL (°)	2.15 ± 0.78	2.88 ± 1.48	0.376	2.6 ± 0.86	3.5 ± 0.1	0.079
Phase angle _ LL (°)	2.18 ± 0.51	2.72 ± 1.33	0.435	3.11 ± 1.42	2.5 ± 0.86	0.716
ECW (L)	14.9 ± 3.9	15.1 ± 1.9	0.931	13.6 ± 2.2	10.8 ± 1.4	0.132
ICW (L)	19.7 ± 4.7	21.8 ± 2.9	0.475	18.7 ± 2.4	15.3 ± 1.1	0.59
ECW ratio	0.43 ± 0.01	0.41 ± 0.01	0.051	0.42 ± 0.01	0.42 ± 0.01	0.657

ECW: extracellular water, ICW: intracellular water, LA: left arm, LL: left leg, POD: postoperative day, RA: right arm, RL: right leg, TR: trunk, WB: whole body, WBC: white blood cell.

a For non-normally distributed variables, non-parametric tests were performed using the Mann–Whitney test.

Interestingly, our results demonstrated a significant negative association between PhA values and other inflammatory markers, but not with WBC counts. WBCs are inflammatory markers, but they are also increased in a variety of conditions, such as allergic disorders, parasitic infections, systemic autoimmune diseases, and aseptic inflammation. This non-specificity of WBCs, an indirect indicator of the degree of inflammation, may have been the reason no significant correlation with PhA values was found. However, other conventional markers including CRP, PCT, and presepsin showed significant associations with PhA. But, in order to obtain laboratory results, conventional inflammatory markers require blood sampling from the patient, and the results take more than an hour to receive. Furthermore, testing for these markers is not available in all healthcare facilities. Therefore, these limitations may reduce their usefulness as markers for the early diagnosis of postoperative infections. In contrast, the PhA on BIA can always be measured in a simple, easy, and non-invasive way, and the results can be obtained in just 5 minutes. As a result, when it is necessary to quickly diagnose postoperative infections, the measurement of PhA using BIA could avoid time-consuming, invasive, and unnecessary blood sampling, and could be used as an indicator to quickly and indirectly assess an infection. Of course, further studies should be conducted for comparing the diagnostic accuracy between the PhA and other inflammatory markers. However, we expect that the PhA on BIA could be an additional tool to monitor and evaluate the development of infections in patients after abdominal surgery with non-invasiveness and simplicity of measurement, and will be more useful for postoperative patients who experience frequent blood draws, time-consuming invasive procedures, and severe wound pain.

In the current study, lower PhA values measured at 50 kHz were identified as a risk factor for postoperative infections in females. Therefore, a low PhA value may help in the early recognition of a developing infection, and the physician can perform additional culture tests to identify the bacterial species and escalate the administration of empirical antibiotics accordingly, and imaging tests, such as computed tomography scans, can be performed early and quickly for a more reliable diagnosis and confirmation of the infection source. Consequently, this may facilitate the drainage of a contaminated fluid collection or surgical treatment of the infection as soon as possible, thereby facilitating the treatment of subsequent infections and improving clinical outcomes. Our results also showed that higher CRP levels measured on POD1 in males, and higher presepsin levels and APACHE II scores in females were predictors of postoperative infections. Using these various risk factors in addition to the PhA measured by BIA will be helpful in the prediction, early recognition, and proper treatment of patients with postoperative infections. However, only risk factors for diagnosing the occurrence of postoperative infections were analyzed in this study, and whether they ultimately affected clinical outcomes, such as mortality, was not analyzed. Therefore, additional research related to the interpretation of the findings is needed. Nevertheless, our study may be helpful in the early diagnosis of postoperative infections through PhA measurement.

The results of the current study should be interpreted with caution due to various limitations. Firstly, as in other previous studies (13, 16, 17, 28), the cross-sectional design of the current study could not allow for identifying causality between PhA values and other inflammatory markers, which limits the external validity for other populations. Therefore, there are inevitable limitations that cannot draw conclusions due to research methodological limitations. Additionally, we retrospectively reviewed and analyzed data from a single institution composed of a relatively uniform race. Based on the independence of the various common confounding variables included in our study, our results could be valid for our populations. However, considering that the standard values for body composition can differ by factors such as race, the reliability, reproducibility, and universality of the results should be demonstrated in a multicenter study based on a large sample that includes various races in the future. Secondly, baseline phase angle values that was known to related to the nutritional status of patients, a risk factor for postoperative infection were not included. As per our institutional policy, our team is performing treatment after surgery and admission to the ICU, there are limitations to performing bioelectrical impedance analysis before surgery. However, the baseline nutrition status was indirectly assessed using the subjective global assessment score, and there was no significantly difference in SGA score between two groups as shown in Table 1. In the next study, the data of baseline phase angle should be included and analyzed to confirm the influence of baseline phase angle value for postoperative infection. (29) And, we actually measured BIA several times such as 36, 72 h postoperatively, but it was not able to conclude in this study because the number of participants analyzed at that time was insufficient to analyze the relationship between phase angle change and clinical outcomes. Authors suppose that additional analysis with a sufficient data of phase angle measured serially should be conducted in the next study for determining the relationship between the change of phase angle and clinical results. Thirdly, bioelectrical impedance vector analysis (BIVA) could not be performed. BIVA is known to detect changes in tissue hydration status or soft tissue mass regardless of body weight. Unfortunately, we did not collect and failed to analyze data using BIVA in the current study. In the near future, BIVA should be analyzed and presented together. Fourthly, we did not analyze some inflammatory markers such as IL-6 or TNF-a, because various important cytokines such as IL-6, TNF-α or IFN-γ are not covered by health insurance in our country, and the cost of testing these cytokines is much higher than that of other conventional markers. Nevertheless, our results included all major inflammatory markers that are most commonly used in clinical practice and are well-known to have significant associations with various pathologic processes. (30) Finally, various factors in the ICU environment, such as skin temperature, ambient air, or seating, that could affect BIA measurements, were difficult to fully control. However, we conducted BIA measurements under specially controlled conditions, including constant temperature and humidity, and we believe that this may have helped to minimize the bias from environmental factors in the ICU. Nevertheless, this study is meaningful in that it differs from previous studies. This was a prospective cohort study of patients who underwent major abdominal surgery. In addition, this study focused on critically ill patients who underwent abdominal surgery, who were particularly vulnerable to infection. So, it is important to predict postoperative infection and to promptly diagnose and treat infection for the patient’s prognosis. However, inflammatory markers used to diagnose infection had the disadvantage of blood sampling from patient and taking more than an hour to get results. Through this our study, we revealed the significant association between inflammatory markers and phase angle, and also the significant differences in the phase angle according to the occurrence of postoperative infections. Phase angle measurement through BIA, which can be performed non-invasively within 5 min, could serve a role as an important and rapid indicator for ICU patient infection treatment. Also, in the case of abdominal surgery, there are massive tissue injury during surgery and the inflammatory response in the abdominal cavity that normally occurs after surgery. Therefore, clinical symptoms that are not truly pathogenic are very common, usually include inflammatory symptoms such as leukocytosis, fever, and abdominal pain. Because of this, it is easy to confuse early postoperative clinical symptoms with early onset of infectious complication results in clinical deterioration. Also, it is difficult to detect the occurrence of infection at an early stage, which delays diagnosis and appropriate examination and treatment, that is a vulnerability that can worsen the prognosis. Therefore, the study results of analyzing the phase angle as a surveillance tool in patients after abdominal surgery, which is more difficult to detect infectious complications, have clinical significance. In particular, this study is distinct from the majority of existing studies that analyzed the relationship between phase angle and infectious complications for nonsurgical patients. So, it is expected to be more useful for patients who are admitted to ICU after surgery.

In conclusion, the phase angle obtained through BIA showed a significant association with inflammatory markers and could be used as a predictor of infections. Phase angle measurements through BIA could improve patient prognosis after abdominal surgery through the careful observation of infections and early, appropriate treatment. Further studies with larger samples through prospective cohort observational study should be needed to clarify our findings drawn in current study.

## Data availability statement

The data that support the findings of this study are available from the corresponding author, upon reasonable request.

## Ethics statement

The studies involving human participants were reviewed and approved by Institutional Review Board of the College of Medicine of the Catholic University of Korea. Written informed consent for participation was not required for this study in accordance with the national legislation and the institutional requirements.

## Author contributions

GL performed literature search, wrote the manuscript, collected data, and performed the statistical analysis. EK collected the data, designed the study, and revised the manuscript. GL and EK helped to perform the research. All authors contributed to the article and approved the submitted version.

## Conflict of interest

The authors declare that the research was conducted in the absence of any commercial or financial relationships that could be construed as a potential conflict of interest.

## Publisher’s note

All claims expressed in this article are solely those of the authors and do not necessarily represent those of their affiliated organizations, or those of the publisher, the editors and the reviewers. Any product that may be evaluated in this article, or claim that may be made by its manufacturer, is not guaranteed or endorsed by the publisher.

## Abbreviations

APACHE II: Acute Physiology and Chronic Health Evaluation II
BIA: Bioelectrical impedance analysis
BIVA: Bioelectrical impedance vector analysis
CI: Confidence interval
CRP: C-reactive protein
ECW: Extracellular water
IC group: Groups with infections
ICU: Intensive care unit
NC group: Groups without infections
OR: Odds ratio
PhA: Phase angle
POD1: Postoperative day 1
SOFA: Sequential organ failure assessment
WBC: White blood cell
PCT: Procalcitonin
